# Ethical governance of artificial intelligence in digital health for Indigenous populations: A narrative review and conceptual framework

**DOI:** 10.1177/20552076261481276

**Published:** 2026-08-22

**Authors:** Amal Khan, Alyson S. N. Bear, Mairyn Rackow, Cassandra Opikokew Wajuntah, Kenneth Lai, Veronica McKinney, John Costa, Ivar Mendez

**Affiliations:** 1 Department of Community Health & Epidemiology, College of Medicine, 7235 University of Saskatchewan, Saskatoon, SK, Canada; 2 736079 Virtual Health Hub, Saskatoon, SK, Canada; 3 Whitecap Dakota Nation Department of Legal Services, Whitecap Dakota Nation, SK, Canada; 4 College of Law, 7235 University of Saskatchewan, Saskatoon, SK, Canada; 5 Saskatchewan Environments for Indigenous Health Research (SK-NEIHR), 7235 University of Saskatchewan, Saskatoon, SK, Canada; 6 Northern Medical Services, College of Medicine, 7235 University of Saskatchewan, Saskatoon, SK, Canada; 7 Department of Surgery, College of Medicine, 7235 University of Saskatchewan, Saskatoon, SK, Canada

**Keywords:** artificial intelligence, digital health, indigenous health, AI ethics, indigenous data sovereignty, health equity, ethical framework

## Abstract

Artificial intelligence (AI) integration into digital health care delivery is rapidly expanding and is increasingly viewed as a promising approach for improving access to care, strengthening diagnostic decision-making, and addressing persistent health inequities experienced by Indigenous populations. Although AI-enabled digital health applications offer potential benefits such as scalability, personalization, and improved clinical decision support, they also raise critical ethical concerns related to equitable implementation, culturally grounded design, representative data usage accountability, privacy protection, and Indigenous data sovereignty. A coherent ethical governance framework is necessary to guide the responsible development, deployment, and evaluation of AI applications implemented in digital health solutions involving Indigenous communities. A narrative review was conducted to critically assess recent literature (2020–2025) addressing ethical considerations in the integration of AI into digital health care delivery for Indigenous populations. Searches were performed in MEDLINE, PubMed, Web of Science, and Scopus using terms related to artificial intelligence, digital health, Indigenous populations, and ethics, with supplementary grey literature searches conducted to identify policy and implementation reports. Studies were selected based on relevance to AI-enabled digital health initiatives implemented in digital health contexts involving Indigenous communities in Canada, the United States, Australia, New Zealand, and the United Kingdom, and were synthesized using thematic narrative analysis. Six themes denoting ethical domains were identified: (1) co-design of AI technologies, (2) culturally grounded implementation, (3) bias and representative data, (4) accountability, transparency, and intelligibility, (5) privacy and Indigenous data sovereignty, and (6) resource accessibility. These domains function as interconnected components of ethical governance, with participatory governance and Indigenous data sovereignty emerging as foundational cross-cutting principles. The proposed framework is not intended as a pan-Indigenous or one-size-fits-all model. Instead, it is conceptualized as a flexible, distinctions-based ethical governance framework that must be adapted to the specific cultural, geographic, and governance contexts of individual Indigenous communities. Based on this analysis, we propose a conceptual ethical governance framework to support policymakers, clinicians, health systems, digital health developers, as well as Indigenous communities in identifying ethically appropriate safeguards across the lifecycle of AI-enabled digital health initiatives planned or implemented in Indigenous communities.

## Introduction

Persistent health inequities continue to affect many populations globally, reflecting structural disparities in access to care, resources, and participation in health system decision-making. Artificial intelligence (AI) is increasingly integrated into digital health care delivery and is widely viewed as a promising strategy for improving health outcomes and strengthening health system performance. AI-powered systems are able to analyze massive data inputs to identify patterns and predict results; they can also assist with accuracy, earlier recognition of health risks, and improvement in patient outcomes.^1^ There is also the promise for this technology to bridge healthcare gaps and meet the unique needs of populations experiencing systemic inequalities in healthcare access^2^ across all levels of care: primary (prevention), secondary (acute) and tertiary (specialized) care. The World Health Organization (WHO) even considers AI to have the potential of “helping all countries achieve universal health coverage”.^3^ Commentary on the potential positive systemic change which AI could introduce in public health is a rapidly growing trend in academic literature.^4,5^

Despite these potential benefits, the integration of AI into digital health delivery also raises important ethical concerns that must be addressed to ensure that technological innovation contributes to health equity rather than reinforcing existing disparities. An equally prevalent sentiment often follows the promotion of AI innovations: the need for AI to be integrated ethically, to both do “good” and contribute to the overall “common good”.^6^ Key ethical considerations include equitable participation in system design, culturally grounded implementation, representative training data, accountability for automated decision-making, privacy protection, and responsible data governance. These concerns are particularly significant in digital health contexts involving Indigenous populations, who experienced barriers accessing health services, exclusion from governance processes, and disproportionate risks associated with misuse of health-related data.^2,7,8^ Without intentionally building Indigenous perspectives, governance structures, and knowledge systems into AI development and deployment processes, digital health innovations risk reproducing or amplifying existing structural and systemic inequities.

Although a growing body of literature discusses ethical considerations related to AI use in health care, there remains no consolidated ethical governance framework to apply these considerations specifically for the integration of AI into Indigenous populations’ digital health delivery. Developing such a framework is necessary to guide policymakers, developers, health systems and the Indigenous communities themselves in identifying ethically appropriate approaches across the design, implementation, and evaluation stages of AI-enabled digital health initiatives. In view of this, it is important to identify what specific ethical considerations need to be incorporated in the integration of AI in digital care for Indigenous populations. At present, there is very limited research on the topic of ethical considerations in the integration of AI in a general sense, and to the best of our search, no research has focused on the potential impacts on Indigenous populations. Accordingly, this narrative review aims to synthesize the existing literature on ethical considerations related to the integration of AI into digital health for Indigenous populations and to develop a conceptual ethical governance framework derived from the key thematic domains identified in the literature. The terms *Indigenous peoples*, *Indigenous communities*, and *Indigenous populations* are used intentionally according to context and are not intended to be used interchangeably. *Indigenous peoples* is used when referring to collective rights, governance, and self-determination, whereas *Indigenous communities* and *Indigenous populations* are used when discussing healthcare implementation contexts or the populations represented within the reviewed literature.

## Methods

### Positionality and reflexivity

This review was conducted by a multidisciplinary team comprising Indigenous and non-Indigenous researchers with expertise in Indigenous health, digital health, artificial intelligence, ethics, law, medicine, and community-engaged research. The author team includes First Nations, Métis, Chinese-Métis, Indigenous Bolivian, and racialized scholars. Indigenous representation within the team includes members of Canoe Lake Cree Nation, Whitecap Dakota Nation, Métis communities, researchers with a decade-long partnership working with Indigenous communities across Saskatchewan, healthcare leaders, clinicians, and legal scholars whose lived experiences and professional expertise informed the interpretation of the literature and refinement of the proposed ethical governance framework. Throughout this work, we sought to uphold the principles of respect, reciprocity, Indigenous self-determination, and “nothing about Indigenous peoples without Indigenous peoples.” These perspectives complemented the evidence synthesized through this structured narrative review.

### Search strategy

To provide a comprehensive overview of ethical considerations relevant to the integration of AI into digital health contexts involving Indigenous populations, a structured narrative review of the literature was conducted^9,10^ using a transparent and reproducible search strategy.^11^ This methodology was chosen because the objective was to synthesize and critically interpret empirical studies, policy documents, governance frameworks, and conceptual literature to inform the development of a conceptual ethical governance framework. The emerging and interdisciplinary nature of this field, together with the heterogeneity of the available evidence, made a **structured narrative review** well suited to the aims of this study. Unlike systematic or scoping reviews, which aim to comprehensively identify or map the available evidence, this structured narrative review was undertaken to critically synthesize and interpret heterogeneous evidence to inform the development of a conceptual ethical governance framework. The search strategy was designed to identify literature relevant to the review objectives across health sciences, bioethics, digital health, and Indigenous health. Multiple databases were searched to capture the interdisciplinary nature of the topic, while supplementary grey literature searches and citation tracking were used to identify relevant policy documents, governance frameworks, and other influential publications not consistently indexed in bibliographic databases. An ethics exemption (E817) was obtained from the University of Saskatchewan Research Ethics Board. Literature searches were conducted between June and July 2025, with final updates completed in August 2025. Searches were performed in MEDLINE, PubMed, Web of Science, and Scopus, selected to capture literature across health sciences, bioethics, digital health, and interdisciplinary research relevant to the review objectives. The search strategy was developed in consultation with the University of Saskatchewan Health Sciences librarian and modified from a search strategy sheet provided by the University of Ottawa.^11^ The librarian trained in literature review guided the development of search terms and terminologies (Supplemental Appendix 1; S1 File). Search strategies were developed using combinations of keywords related to “artificial intelligence,” “digital health,” “Indigenous populations,” and “ethics” (Supplemental Appendix 1; S2 File), with supplementary grey literature searches conducted to identify policy documents and governance frameworks (Supplemental Appendix 2, S3 File). The searches were organized around three main areas of ethical relevance: 1. Ethical considerations related to AI in health care and digital health delivery, 2. Ethical governance, Indigenous data sovereignty, and community-based health data stewardship, and 3. Ethical considerations for digital health technologies implemented in contexts involving Indigenous communities. The review focused on literature from Canada, the United States, Australia, New Zealand, and the United Kingdom because these countries have well-established digital health systems and published AI governance initiatives relevant to the review objectives. The United Kingdom was included because of the relevance of its digital health policy and AI governance literature, particularly guidance developed through the National Health Service (NHS), as well as its applicability to broader discussions of AI-enabled digital health involving diasporic Indigenous populations.

### Article selection

Included publications addressed ethical considerations related to the integration of AI in digital health contexts involving Indigenous populations, including ethical principles, governance frameworks, policy analyses, empirical studies, and conceptual evaluations. Full-length articles were eligible for inclusion. Articles were excluded if they were unavailable in full text, published in a language other than English, or did not explicitly address ethical, governance, or implementation considerations related to AI-enabled digital health involving Indigenous populations. Given the breadth of the topic and variability in how ethical considerations are addressed across the literature, article selection combined formal search results with identification of additional key publications through citation tracking of included articles and grey literature searches. Titles and abstracts were screened for relevance, and a full-text review was conducted for eligible studies using an application, Rayyan, which was used to facilitate the organization and documentation of the screening process.^12^ Because this is a structured narrative review, the goal was not to exhaustively identify every eligible publication, but to synthesize literature meeting the predefined eligibility criteria and aligned with the review objectives to inform the thematic analysis and development of the proposed ethical governance framework.

**Figure 1.**
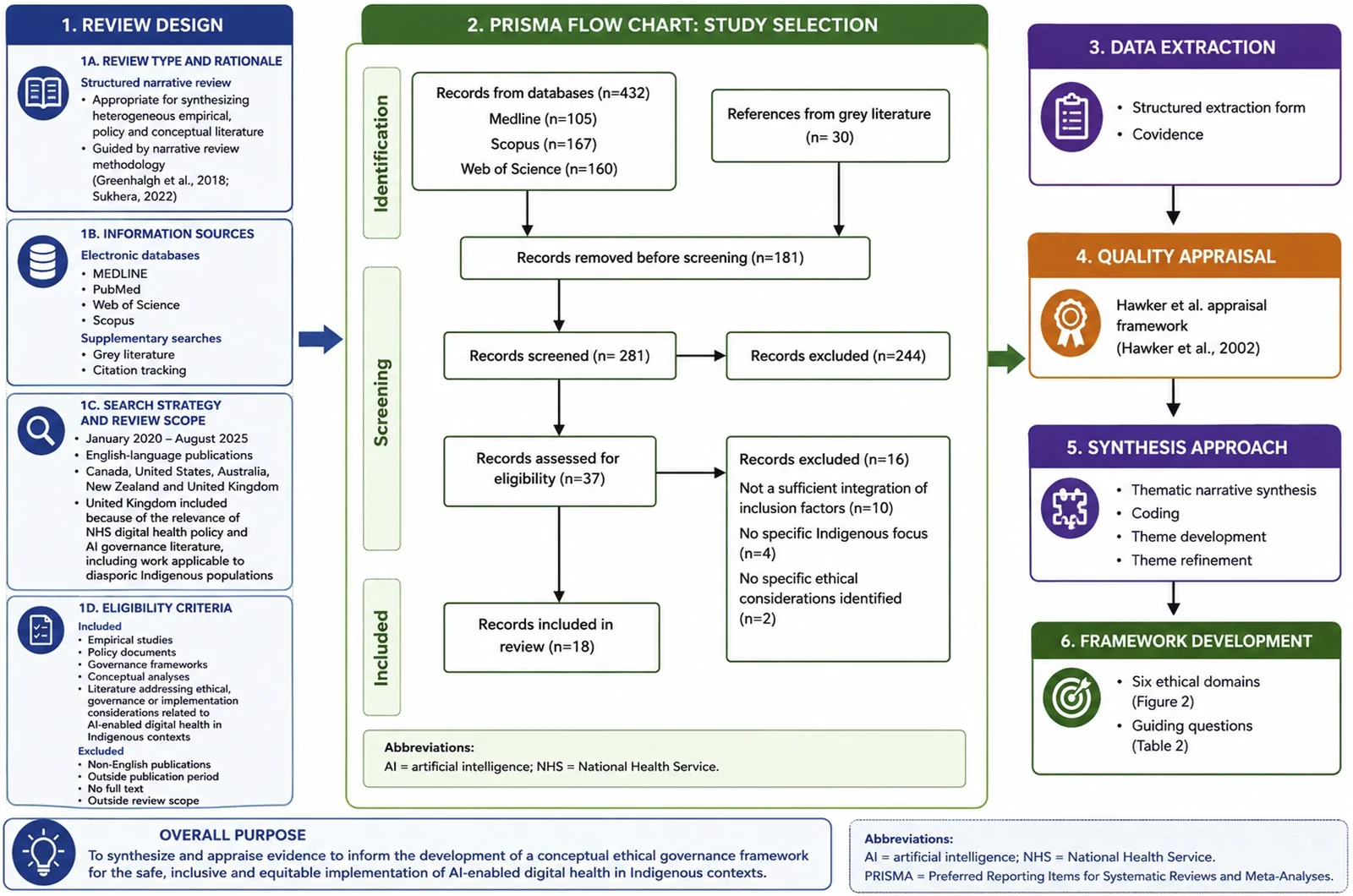
PRISMA-style flow diagram of the literature search and study selection process.

Initial screening and selection were conducted by one author (M.R) under the close supervision of the lead author (A.K.) throughout the review process. Although the primary screening was conducted by M.R., A.K. provided ongoing oversight and guidance during study selection. Also, a subset of studies with uncertain eligibility was independently screened by the lead author (A.K.). Any discrepancies in screening decisions were identified through comparison of independent assessments and resolved through discussion and consensus. This process helped ensure consistency in the application of the eligibility criteria throughout the screening process. Final inclusion decisions were subsequently reviewed with the supervising investigators. Covidence was used to facilitate a full-text eligibility review and data extraction into a structured extraction form. Articles were also assessed for quality using Covidence (Supplemental File Appendix 3; S5 File); using the Hawker et al.^13^ appraisal framework because it permits the assessment of diverse forms of evidence using a common set of quality domains. Where appraisal domains were not applicable to policy documents, guidance reports, or conceptual papers, they were recorded as not applicable. Quality appraisal was undertaken to characterize the methodological quality of the included literature and inform interpretation of the findings; studies were not excluded based on appraisal outcomes. Overall, eighteen articles were included in the final synthesis, representing the three identified areas of ethical relevance. Figure 1 provides an overview of the structured review process.

### Data analysis

A thematic analysis was conducted to synthesize ethical considerations identified across the included literature. The analysis was guided by Braun and Clarke’s thematic analysis approach and employed a combined inductive and deductive coding strategy. Coding focused primarily on semantic themes, capturing ethical concepts explicitly reported in the included literature while allowing additional themes to emerge through iterative analysis. Initial coding and thematic development were conducted by authors (M.R. in consultation with A.K.), who coded the ethical content of the included articles. These codes were organized into overarching themes and subthemes, which were iteratively refined through discussions among the research team (M.R., A.K, and I. M), with additional input from all co-authors (A.B, C.O.W., V.M., K.L., and J.C). The identified themes were subsequently synthesized into a conceptual ethical governance framework developed collaboratively by authors (A.K., M.R., A.B., and I.M.) and discussed with other authors (V.M., C.O.W., K.L., and J.C) for input and refinement. The framework integrates the thematic findings into a structured set of ethical considerations relevant to the design, implementation, and evaluation of AI-enabled digital health initiatives involving Indigenous populations. The proposed framework is conceptually informed by recurring ethical domains identified across the reviewed literature and is intended to support ethical reflection and governance deliberation.

## Results

We identified six main ethical themes, each with associated subthemes. The six themes are as follows: (1) co-design of AI technologies, (2) culturally grounded implementation, (3) bias and representative data, (4) accountability, transparency, and intelligibility, (5) privacy and Indigenous data sovereignty, and (6) resource accessibility. Table 1 provides a short description of the subthemes, along with a list of the literature in which each subtheme was identified.

**Table 1.** Summary of identified subthemes with brief descriptions and the literature sources in which each subtheme was identified.

Theme	Subtheme	Description	References
Co-Design of AI Technologies	Participatory Co-Design	Active involvement of Indigenous communities in AI system planning, development, and evaluation to ensure alignment with community priorities and knowledge systems	^2,4,5,14–25^
	Diverse Development Teams	Inclusion of culturally diverse, anti-colonial, and representative leadership within AI development teams to reduce bias and support inclusive innovation	^4,14,16–18,20,24,26^
Culturally Grounded Implementation	Cultural Appropriateness	Implementing AI systems in ways that reflect Indigenous cultural values, trauma-informed practices, and community-defined priorities	^2,4,5,17–20,22,23,26–28^
	Linguistic Accessibility	Ensuring AI tools do not exacerbate language barriers and are contextually relevant to Indigenous populations	^2,4,16,18,20,23,26–28^
Bias & Representative Data	Algorithmic and Structural Bias	Identifying and mitigating algorithmic, selection, and data biases that may reinforce health inequities or cause patient harm	^21–25,27–29^
	Representative Data	Ensuring datasets reflect Indigenous populations to promote accuracy, reduce inequity, and prevent fabricated or distorted outputs	^5,14,17–19,21,24–29^
Accountability, Transparency and Intelligibility	Accountability Mechanisms	Establishing clear responsibility, remedy, and oversight structures for AI system deployment in Indigenous health settings	^5,17,19,24,26^
	Transparency	Communicating AI system properties, data use, and limitations to support trust and oversight	^2,5,14,17–19,22,24–26^
	Intelligibility	Presenting AI-related information in understandable forms for healthcare professionals and affected communities	^14,15,21,30^
Privacy and Indigenous Data Sovereignty	Privacy Protection	Safeguarding individual and community health data through consent, anonymization, and secure governance	^14,16,18–27^
	Indigenous Data Sovereignty	Upholding collective rights of Indigenous peoples to own, control, access, and govern data derived from their communities	^2,5,16–18,20,25,31^(p.5.)^^
Resource Accessibility	Infrastructure and Affordability	Addressing technological infrastructure, affordability, and system capacity constraints affecting AI implementation	^14,16,17,20,23,24,27^
	Digital Training	Building skills and capacity among Indigenous communities and healthcare professionals to support AI governance and implementation	^2,4,14,17,19,20,23,26,27,32^

### Theme 1: Co-design of AI technologies

#### Subtheme 1.1: Participatory Co-Design

Participatory co-design requires that Indigenous communities be actively involved in the planning, development, and evaluation of AI-enabled health technologies so that these systems reflect community priorities, cultural values, and Indigenous knowledge systems. Such involvement is necessary to ensure that the introduction of AI technologies does not reproduce structural inequities that may arise when technologies are developed without community participation. Fifteen of the 18 included articles^2,15,23^ emphasized the importance of involving Indigenous populations in the design stage of AI systems. WHO highlighted the importance of AI technologies being developed and evaluated with active participation from the populations impacted by them,^26^ and relevant actors are encouraged to prioritize Indigenous-led AI design.^16^ Meaningful relationships must be deliberately formed to ensure Indigenous involvement at every stage of AI development.^17,18^ Ensuring that Indigenous communities retain meaningful authority over the design and implementation of AI systems has been identified as particularly important in contexts shaped by longstanding experiences of structural discrimination in health systems.^21^ Embedding Indigenous knowledge systems within the design process was identified as a key mechanism for promoting equitable technological outcomes^5,18^ during development, which increases the potential benefits of AI technologies.^20^

#### Subtheme 1.2: Diverse development teams

Diverse development teams help ensure that multiple cultural, disciplinary, and lived-experience perspectives inform the design and implementation of AI-enabled health technologies. In AI development, a lack of diversity among those building systems can contribute to biased assumptions, inequitable design choices, and downstream algorithmic bias that may disadvantage historically marginalized populations. Several articles^14,24^ emphasized the importance of including diverse perspectives, including anti-colonial and anti-racist perspectives, in AI development teams.^20^ The inclusion of diverse perspectives was also identified as a crucial component of developing inclusive AI.^4^ Lack of diversity in development teams has been identified as a root cause of algorithmic bias,^18^ supporting calls to diversify teams as part of efforts to “decolonize AI”.^18^ Related governance guidance, including the CARE statement, further reinforces the importance of Indigenous leadership in data and technology development impacting Indigenous communities.^16^ The New Zealand Ministry of Health additionally highlighted the need for culturally grounded leadership in the design and implementation of AI systems.^17^ Ensuring representation not only in participation but also in leadership roles is therefore positioned as a key requirement for ethically responsible AI system development.

### Theme 2: Culturally grounded implementation

#### Subtheme 2.1: Cultural appropriateness in implementation

Culturally grounded implementation refers to the adaptation of AI-enabled digital health technologies to reflect the cultural values, social contexts, and lived realities of the Indigenous communities in which they are deployed. Ensuring cultural responsiveness is essential for preventing technological interventions from reproducing cultural barriers that limit access, trust, and sustained engagement with digital health services. Studies^5,23,28^ emphasized that implementation strategies must recognize the unique cultural needs and contexts of Indigenous populations. Relevance and accessibility to specific Indigenous populations must be considered when introducing AI technologies,^2^ and culturally grounded approaches increase meaning for relevant populations and address cultural barriers.^22^ Similarly, culturally safe and trauma-informed AI approaches were linked with more favourable outcomes,^20^ while the incorporation of traditional Indigenous knowledges into care delivery was identified as improving cultural responsiveness.^5^ Ensuring that cultural responsiveness is defined by Indigenous communities themselves was consistently identified as central to ethical implementation.^22^

#### Subtheme 2.2: Linguistic and contextual accessibility

Linguistic and contextual accessibility refers to the requirement that AI-enabled digital health technologies be implemented in ways that reflect the language needs and communication contexts of the Indigenous communities in which they are deployed. If AI tools are introduced without consideration of language compatibility and local healthcare realities, they risk reinforcing existing access gaps rather than bridging them. Several studies^4,16,28^ identified the importance of ensuring that AI technologies do not further language barriers and are relevant to the populations they are intended to serve. AI tools should therefore be implemented in ways that explicitly address linguistic accessibility and avoid widening communication gaps. It was further noted that implementation strategies should prioritize bridging existing healthcare inequities^24^ rather than merely introducing new technologies. Governance guidance also emphasized that implementation must align with Indigenous health system priorities and community-defined expectations regarding service delivery and data use.^17^ Ensuring linguistic and contextual alignment was therefore identified as a necessary condition for ethically responsible AI deployment in Indigenous health contexts.

### Theme 3: Bias & representative data

#### Subtheme 3.1: Algorithmic and structural bias

Bias in AI-enabled health technologies refers to systematic errors arising from data, design, or deployment processes that produce inequitable or inaccurate outcomes for certain populations. In digital health contexts involving Indigenous communities, biased systems risk reinforcing existing health inequities. Studies^18,28^ discussed the importance of identifying and minimizing bias in AI technologies. Multiple forms of bias were identified across stages of AI integration, including algorithmic bias,^18^ selection bias,^14^ and data collection bias.^21^ Regardless of type, biased AI systems risk further entrenching existing health inequities^17,24^ and may result in misinterpretation of patient data, leading to potential patient harm.^4^ The importance of having systems that explicitly acknowledge potential bias and actively work to reduce its presence was identified as an important consideration for recognizing and addressing bias.^18^ Regular assessment of AI systems to identify and mitigate bias was recommended,^19^ and validation of systems trained on non-Indigenous populations before use in Indigenous healthcare settings was advised.^25^ One study further cautioned that biased AI outputs could shift public health focus away from addressing underlying social and structural determinants of health toward narrow disease control.^14^ Addressing bias was therefore identified as a critical requirement throughout the AI development and implementation lifecycle.

#### Subtheme 3.2: Representative data

Representative data refers to the inclusion of sufficient and appropriate data reflecting the populations within which AI-driven technologies are responsibly implemented. Because AI systems rely on training and validation datasets for inference and generate predictions and recommendations, inadequate representation of Indigenous populations in training and validation datasets may compromise both accuracy and equity. Studies^17,18,28^ emphasized the importance of ensuring that data used in AI systems are properly representative of the communities in which they will be deployed. Frameworks prioritizing representative data from relevant populations were recommended,^24^ and the robustness of AI algorithms was identified as dependent on the degree of representative training data received.^28^ Inclusion of representative data was cited as necessary to ensure equitable outcomes for underrepresented populations.^29^ RANZCR recommended that training dataset composition, including representation of underrepresented populations, be clearly reported to facilitate appropriate implementation of AI tools in healthcare.^25^ Limited datasets reflecting existing health inequities were noted to perpetuate and potentially worsen disparities when used in decision-making,^14^ underscoring the need for deliberate and explicit inclusion of Indigenous populations in AI training datasets.^18^ Inadequate training data was also identified as a contributing factor to fabricated outputs produced by AI tools.^14^ Ensuring representative data was therefore identified as a central strategy for mitigating bias and promoting equitable AI deployment in Indigenous health contexts.

### Theme 4: Accountability, transparency, and intelligibility

#### Subtheme 4.1: Accountability mechanisms

Accountability in AI-enabled digital health refers to the clear assignment of responsibility for system design, deployment, performance, and potential harms. In the context of AI systems implemented in Indigenous health settings, accountability is essential to ensure that decision-making authority and responsibility are not obscured by technological complexity. Studies^24,26^ highlighted the importance of ensuring accountability in the implementation and use of AI technologies. The New Zealand Ministry of Health emphasized designing data ecosystems that benefit Māori populations while ensuring that those responsible for system design remain accountable to these communities.^17^ WHO identified the creation of appropriate accountability mechanisms to assure remedy and redress as a core principle of ethical AI governance.^26^ It was also noted that patient confidence may be strengthened when there is a clearly identified point of responsibility for AI-informed decision-making.^19^ Establishing explicit accountability structures was therefore identified as a necessary safeguard in the ethical deployment of AI-enabled digital health systems.

#### Subtheme 4.2: Transparency

Transparency refers to the clear communication of how AI systems function, how data are collected and used, and how decisions are generated. Because AI systems elude empirical understanding, they practically operate as a “black box”; their limited transparency may undermine trust and reduce meaningful oversight. Studies^17,18^ identified transparency as an ethical consideration. Limited transparency was noted to reduce both trust and accountability in the use of AI in digital health.^24^ A particular need to demonstrate transparency to Indigenous populations was emphasized in light of historical injustices and valid skepticism about health systems.^2,22^ The “black box” nature of AI systems was described as a challenge in understanding specific reasoning behind AI outputs.^14,28^ WHO recommended documenting relevant information about AI technologies, including system properties, limitations, and development processes, both prior to deployment and following approval.^26^ Practicing transparency was linked to increased trust and was deemed foundational to establishing and maintaining effective collaboration with Indigenous communities.^17,18^ Transparency was therefore positioned as central to building trust and supporting accountable AI governance.

#### Subtheme 4.3: Intelligibility

Intelligibility refers to the requirement that information about AI systems, including their functioning, outputs, and limitations, be communicated in ways that are understandable to those affected by their use. In digital health contexts, transparency alone may be insufficient if information is presented in technical or inaccessible formats that limit meaningful comprehension. Several included articles^5,21^ emphasized the need for AI-related information to be reported in ways that are understandable to healthcare professionals and the public. The “black box” nature of AI was identified as creating risks that system reasoning and outputs may not be adequately accessible or interpretable.^14^ WHO advised that AI information be reported both transparently and in an intelligible manner, specifying that systems should be understandable “according to the capacity of those to whom they are explained”.^26^ RANZCR highlighted the importance of ensuring that healthcare professionals can understand and appropriately interpret information pertaining to AI tools.^25^ Intersectoral collaboration was identified as a potential mechanism to support capacity building and knowledge exchange between AI developers and health professionals.^14^ Ensuring intelligibility was therefore identified as essential for enabling informed interpretation, responsible use, and meaningful oversight of AI technologies in Indigenous health contexts.

### Theme 5: Privacy and indigenous data sovereignty

#### Subtheme 1: Privacy protection

Privacy refers to the protection of individual and community health information from misuse, unauthorized access, or inappropriate secondary use. In AI-enabled digital health systems, privacy concerns are amplified because large volumes of sensitive data are collected, stored, and processed over extended periods. Privacy was discussed as a central ethical consideration.^23,25^ The CARE statement highlighted the importance of strong AI privacy standards to prevent misuse of Indigenous information.^16^ Privacy protections may include anonymization or deidentification techniques, as well as strict access control over sensitive data.^19^ Valid informed consent was identified as essential, requiring that individuals understand what data are collected, how they are stored, and who can access them.^22^ Privacy concerns were also noted to deter participation in data collection, potentially increasing underrepresentation and contributing to biased outcomes.^14^ Implementation approaches that proactively anticipate privacy considerations were identified as facilitating responsible AI deployment in health contexts.^20^ Confidentiality breaches were described as carrying significant social repercussions in rural American Indian or Alaska Native communities, potentially intensifying valid skepticism in health systems.^22^ Privacy protection was therefore positioned as a foundational safeguard in the ethical implementation of AI technologies in Indigenous health settings.

#### Subtheme 2: Indigenous data sovereignty

Indigenous data sovereignty refers to “the rights of Indigenous peoples to own, control, access, and possess data that derive from them and pertain to their members, knowledge systems, customs, or territories”^31(p.5.)^ Unlike individual privacy protections, Indigenous data sovereignty emphasizes collective authority and community governance over the collection, use, and stewardship of data generated through AI-enabled digital health systems. Indigenous data sovereignty was emphasized as a distinct ethical domain.^16^ Because AI systems both use and generate substantial volumes of health data, governance structures must ensure that Indigenous data remains under Indigenous control.^2^ Historical exploitation and appropriation of Indigenous data were identified as reinforcing the need for sovereignty-based governance protections.^16^ Prioritizing Indigenous data sovereignty practices was deemed essential in light of deep-rooted, valid skepticism of healthcare systems among Indigenous populations.^5^ Community-led governance of AI systems was identified as a key strategy for safeguarding data sovereignty and maintaining trust.^2^ The New Zealand Ministry of Health further emphasized modernizing consent processes and enabling systems compliant with Māori principles^17^ as well as supporting local-level data governance to strengthen community authority and oversight.^17^ Indigenous data sovereignty was therefore positioned as a collective governance framework extending beyond privacy to encompass ownership, control, and long-term stewardship of Indigenous health data.

### Theme 6: Resource accessibility

#### Subtheme 1: Infrastructure, affordability, and implementation capacity

Resource accessibility refers to the practical conditions required for AI-enabled digital health technologies to be meaningfully available and usable, including infrastructure, affordability, and health system capacity. Although AI technologies are often positioned as tools to improve access to care, unequal technological resources may limit benefits and may exacerbate inequities if implementation conditions are not addressed. Several articles^14,17^ noted that increased infrastructure is required to make AI-enabled access meaningful in communities experiencing historical marginalization and geographic barriers. Affordability of AI-related resources was identified as a relevant factor in addressing historical health inequities.^23,24^ Technological access and connectivity needs were also highlighted as important considerations.^20^ Indigenous communities were described as often facing limited staffing and finances,^27^ and the CARE statement identified a lack of resources and infrastructure as prohibitive to Indigenous-led AI development.^16^ The New Zealand Ministry of Health further emphasized the importance of assessing the value of new technologies, noting that in some contexts, funding other programs may bridge equity gaps more meaningfully.^17^ Resource accessibility was therefore identified as a necessary condition for equitable implementation of AI-enabled digital health initiatives.

#### Subtheme 2: Digital training

Digital training refers to building the skills and capacity required for Indigenous communities and healthcare workers to engage with, implement, and govern AI-enabled digital health technologies. Without training and capacity building, AI systems may remain inaccessible in practice and may limit the meaningful participation of Indigenous communities across the AI lifecycle. A specific resource solution emphasized across the literature was the provision of digital training for both Indigenous populations and healthcare workers operating in their communities.^23,26^ WHO proposed “sustainable” integration of AI, defining sustainability in this context as anticipating the need for training in AI technologies.^26^ Digital training was also described as needing to be context-specific and to incorporate community perspectives.^27^ Such training was positioned as a mechanism to validate and empower underrepresented populations, with the goal of creating opportunities for Indigenous communities to develop and direct innovation in digital health.^23^ This idea was echoed by the New Zealand Ministry of Health’s emphasis on lifting “capacity and capability” across the health workforce.^17^ Digital training was therefore identified as a key strategy for closing resource gaps and enabling more meaningful Indigenous involvement in AI development, implementation, and governance.

## Discussion

The purpose of this review was to identify ethical considerations relevant to the integration of AI-enabled technologies within digital health contexts involving Indigenous populations, to inform the development of an ethical governance framework. This discussion interprets how the identified themes intersect and their implications for ethical AI implementation in Indigenous health contexts. Our findings highlight the layered ethical complexity that arises at the intersection of AI technologies and Indigenous health governance. Although the themes were presented individually for analytic clarity, they are deeply interrelated. Continual inclusion of Indigenous perspectives is necessary across all stages of AI development and implementation.^18^ The inclusion of diverse perspectives within development teams has been linked to minimizing bias and improving system fairness.^19,26^ Privacy protections are directly connected to Indigenous sovereignty over data^16,25^ and strengthening technological and social infrastructure enables AI tools to be co-designed with Indigenous populations.^16,17,23^ These interconnections illustrate that ethical AI implementation cannot be addressed through isolated safeguards but requires coordinated and overlapping governance mechanisms.

Indigenous data sovereignty and bias mitigation emerged as particularly foundational concerns. Foundational frameworks including the United Nations Declaration on the Rights of Indigenous Peoples (UNDRIP),^33^ the World Intellectual Property Organization (WIPO) treaty on intellectual property,^34^ and guiding principles, including Ownership, Control, Access, and Possession (OCAP),^35^ Collective Benefit, Authority to Control, Responsibility, Ethics (CARE),^36^ and Findable, Accessible, Interoperable, and Reusable (FAIR)^37^ data principles, provide guardrails to ensure ethical AI implementation. These frameworks should also incorporate the Tri-Council Policy Statement, Chapter 9 guidelines,^38^ which require community engagement, reciprocity, and respect for Indigenous governance when research or data involves First Nations, Inuit, and Métis peoples. Accordingly, these principles should be mapped directly onto AI data collection, model evaluation, and field deployment processes. Indigenous data sovereignty extends beyond individual privacy protections by emphasizing collective ownership, control, and stewardship of data^25,31(p.5.)^Given the historical exploitation and appropriation of Indigenous data, sovereignty-based governance is essential to building and maintaining trust in digital health systems.^5,16^ Algorithmic and structural bias present significant risks in AI-enabled systems. Continuous testing, validation, and monitoring have been recommended to minimize bias and ensure adequate representation of Indigenous populations in AI training datasets.^18,19,21^ Without deliberate mitigation strategies, AI systems risk reinforcing existing inequities rather than alleviating them.^4,14,17^ These concerns are further amplified as generative AI and large language models (LLMs) become integrated into digital health care.^39^ While these technologies offer considerable opportunities to enhance clinical decision support, patient engagement, and access to information, they also introduce new ethical challenges, including inaccurate or fabricated outputs, cultural misrepresentation, inappropriate use of Indigenous data and knowledge, and limited transparency in how AI-generated recommendations are generated and applied.^39^ Explainable AI (XAI) may help address some of these challenges by improving the interpretability of AI systems and strengthening transparency, accountability, and trust among clinicians, patients, and Indigenous communities.^40^ Collectively, these challenges demonstrate that AI integration in Indigenous health contexts demands ethical scrutiny beyond traditional public health frameworks.

Co-design and Indigenous-led development represent practical mechanisms for addressing these risks. AI systems should be developed in partnership with those who will use and be affected by them.^26^ Indigenous-led development has been emphasized as critical, as Indigenous communities themselves are best positioned to identify relevant needs and priorities.^16^ This approach may reduce the risk of cultural misinterpretation or appropriation associated with integrating Indigenous knowledge into AI systems.^18^ In jurisdictions such as Canada, these considerations also carry legal significance: Indigenous peoples hold constitutionally recognized rights under section 35 of the Constitution Act, 1982, and many Nations increasingly assert jurisdiction over data governance, health services, and digital infrastructure within their territories. As AI-enabled digital health systems expand, data ownership and stewardship increasingly intersect with Indigenous jurisdiction and self-determination, highlighting that Indigenous data sovereignty reflects not only ethical concerns but also constitutionally protected governance authority. As articulated by Julia A. Silano, the integration of new technologies must honour the ethical principle of “nothing about [Indigenous populations] without [Indigenous populations]”,^4^ ^(p.1.)^ This principle captures the ethical core of responsible AI implementation in Indigenous health contexts.

AI systems also introduce a governance challenge that extends beyond initial implementation. Developments in AI and associated ethical concerns evolve rapidly, requiring continuous consultation, verification, and shared decision-making rather than one-time oversight. The New Zealand Ministry of Health emphasized the importance of maintaining “ongoing and active partnership” to protect Māori data sovereignty.^17^ This provides a practical example of how Indigenous governance principles can be embedded within national digital health policy to support ethical AI implementation.^17^ Similarly, Indigenous digital health initiatives in Canada have highlighted the importance of sustained community partnerships, shared decision-making, and culturally grounded governance in supporting the successful implementation of digital health technologies.^2,5^ Together, these examples illustrate that ethical governance is a practical prerequisite for responsible AI-enabled digital health implementation.

WHO advised that AI technologies should undergo regular testing, monitoring, and evaluation,^22^ and continuous assessment has been associated with minimizing bias and ensuring adequate representation in training data.^18,19,21^ Such ongoing oversight also requires mechanisms for independent accountability and clear pathways for model redress when harms, inaccuracies, or inequities are identified. Ensuring cultural safety similarly requires ongoing relationship building with the populations for whom the technologies are designed.^22^ Ethical governance of AI must therefore be understood as a sustained and adaptive process, not a one-time regulatory checkpoint.

Although there is significant heterogeneity among Indigenous populations,^22^ the overarching concepts identified in this review provide a foundation for ethical reflection. The proposed framework is conceptual and is intended to support ethical deliberation and governance rather than serve as a validated implementation model. It is derived from the available literature and should therefore be viewed as an initial foundation for future empirical evaluation, implementation research, and community-led refinement across diverse Indigenous contexts. This review does not capture every concern across all Indigenous contexts; instead, it identifies cross-cutting themes emerging in the literature to date. In jurisdictions such as Canada, where Indigenous rights may have constitutional implications,^41^ integrating Indigenous perspectives into AI governance may carry not only ethical but legal significance. These considerations underscore the importance of developing a structured framework capable of guiding ethically responsible AI implementation while remaining responsive to local context and community-defined priorities. The proposed framework builds upon established frameworks and principles, including OCAP, CARE, FAIR, UNDRIP, and the WHO guidance on the ethics and governance of AI for health. While these provide important foundations for Indigenous data governance, research ethics, human rights, and trustworthy AI, they primarily address specific ethical or governance dimensions in isolation. The novelty of the proposed framework lies in integrating these complementary principles within a single governance framework tailored to AI-enabled digital health involving Indigenous populations. In doing so, it positions Indigenous data sovereignty, participatory governance, and cultural safety as cross-cutting considerations that inform the entire AI lifecycle—from design and development through implementation, monitoring, and evaluation.

### Creating an ethical framework

**Figure 2.**
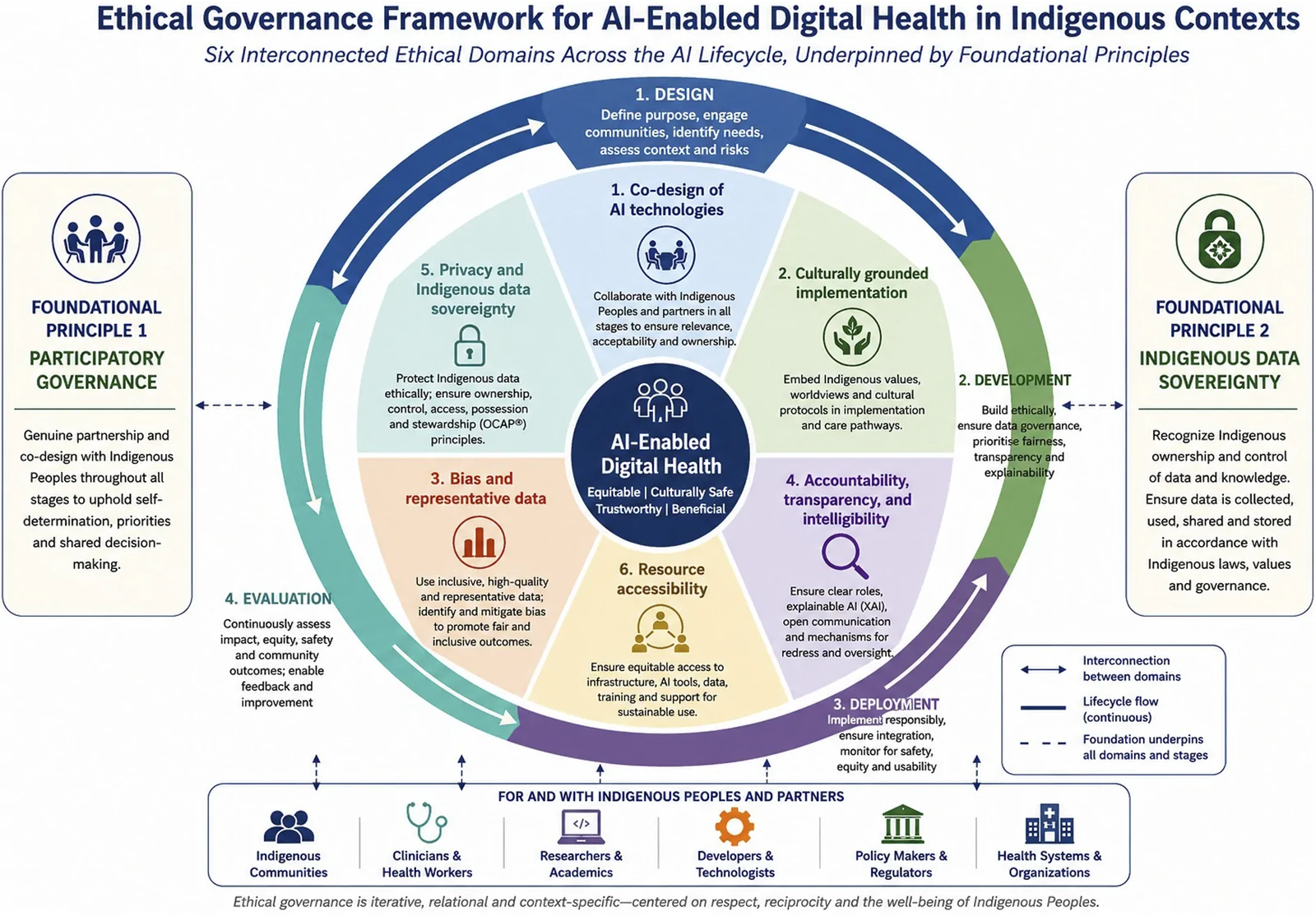
Ethical governance framework.

The findings of this review indicate that ethical governance of AI-enabled digital health systems in Indigenous contexts requires more than reference to general bioethical principles. While equity, accountability, respect, and prevention of harm remain foundational, the integration of AI introduces additional considerations related to algorithmic bias, collective data governance, cultural safety, and infrastructure disparities. These considerations must be articulated in ways that are specific to Indigenous health contexts and responsive to the operational realities of AI-enabled systems. The framework presented in Figure 2 is derived directly from the thematic synthesis of this review and is organized into six interrelated domains that reflect ethical priorities consistently identified across the included literature. These domains are operationalized through **structured guiding questions** intended to support decision-making across design, implementation, and long-term oversight, as given in Table 2. Considered collectively, they provide a practical structure for embedding ethical reflection into ongoing AI-enabled digital health initiatives, assisting researchers, clinicians, developers, Indigenous governance partners and ultimately the Indigenous communities themselves in navigating implementation responsibly.

**Table 2.** Guiding questions: Ethical framework for AI-Enabled digital health in indigenous contexts.

Domain	Guiding questions	Examples of ethical implementation
Indigenous Governance and Data Sovereignty	●How are Indigenous collective rights to own, control, access, and steward data upheld in accordance with Indigenous data sovereignty principles? ●How is governance structured to ensure Indigenous authority over data collection, storage, use, and interpretation? ●How does the initiative address the exploitation of Indigenous data and associated mistrust in health systems? ●How are consent and governance processes aligned with community-defined principles and expectations?	●Signed data-sharing agreement co-developed with the community ●Indigenous-directed and Community-led data governance and stewardship ●Community-defined consent processes
Co-Design and Indigenous Leadership	● How are Indigenous communities meaningfully involved in the design, implementation, and evaluation of the AI-enabled system? ● How are development teams structured to include diverse and representative perspectives that reduce bias? ● How are Indigenous knowledge systems respected and reflected in AI design processes? ● • How does the initiative honour the principle of “nothing about [Indigenous populations] without [Indigenous populations]”?	● Documented community advisory board involvement ● Deliberate Indigenous leadership throughout governance and decision-making ● Intentional co-design throughout the AI lifecycle
Bias and Representative Data	● What potential sources of bias (algorithmic, selection, data collection, structural) are identified across the AI lifecycle? ● How does the system explicitly acknowledge and mitigate potential bias? ● How are AI systems validated locally before use in Indigenous contexts, particularly if trained on external datasets? ● How is ongoing monitoring conducted to identify inequitable outcomes after implementation? ● How is dataset composition transparently documented to support responsible deployment?	● Local validation metrics on a representative subset ● Representative Indigenous training datasets ● Deliberate bias monitoring throughout implementation ● Documented bias audit and mitigation log ● Ongoing reflexive review and improvement log
Accountability, Transparency, and Intelligibility	● How are trade-offs made openly and justified transparently to affected communities? ● How are system limitations, properties, and intended uses documented and disclosed? ● How are transparency practices linked to building and maintaining trust in Indigenous contexts shaped by systemic injustice? ● How is information about the AI system communicated in ways that are intelligible to healthcare professionals and Indigenous communities?	● Clearly defined & discussed governance and accountability ● Transparent documentation of AI use and limitations ● Accessible communication throughout implementation ● Plain-language explanations co-developed with Indigenous communities ● User-tested communication materials for clinicians and communities
Privacy Protection	● How is informed consent obtained for AI system use, data collection, and data sharing? ● How are privacy safeguards (e.g., access control, deidentification) implemented appropriately? ● How are collective privacy considerations addressed where impacts extend beyond individual risk? ● How are privacy risks anticipated and mitigated during implementation planning?	● Community-defined consent procedures ● Privacy protections embedded across data collection, storage, access, sharing, and secondary use ● Privacy impact assessment completed before implementation ● Designated Indigenous data custodians with documented access controls
Resource Accessibility and Capacity Building	● How are infrastructure, affordability, connectivity, and workforce capacity addressed before deployment? ● How is the value of the AI-enabled system assessed relative to alternative interventions that may bridge equity gaps more effectively? ● How is digital training provided to Indigenous communities and healthcare workers? ● How is training tailored to community perspectives and context-specific needs? How does capacity building support Indigenous leadership in innovation rather than passive technology adoption?	● Documented infrastructure and workforce readiness assessment ● Context-directed digital training ● Intentional capacity building throughout implementation ● Structured mentorship and return-of-service pathways for Indigenous trainees

### How to use the framework: Applying the framework in practice

The framework is intended to function as a structured guide for ethical deliberation across the lifecycle of AI-enabled digital health initiatives. It should be used during early conceptualization, revisited during development and validation, and reassessed throughout implementation and long-term evaluation. Applying the framework at multiple stages ensures that ethical considerations are integrated into operational decision-making rather than addressed retrospectively. In practical terms, applying the framework involves working collaboratively through each domain with relevant stakeholders, including Indigenous governance partners, to identify potential risks, clarify accountability structures, and document how ethical priorities are being addressed. Because the six domains are interrelated, reflection within one area (for example, bias mitigation) often requires consideration of others, such as data sovereignty, transparency, or infrastructure readiness. These domains collectively strengthen ethical oversight and reduce the risk of siloed decision-making. The framework is not intended to replace community governance processes or institutional ethics review. Instead, it complements these processes by providing a consistent structure for identifying context-specific ethical considerations that arise in AI-enabled digital health care delivery for Indigenous populations.

### Navigating ethical tensions

AI-enabled digital health systems inevitably generate situations in which ethical considerations intersect and, at times, conflict. The framework supports transparent identification and management of these tensions rather than assuming they can be eliminated. For example, enhanced personalization of digital tools may require expanded data collection, intensifying privacy and data sovereignty considerations. Efforts to scale systems across regions may improve efficiency while challenging local adaptation and cultural specificity. Predictive modelling may enhance early intervention while requiring careful monitoring to prevent biased outcomes. By making these tensions explicit, the framework enables stakeholders to articulate trade-offs transparently, ensure Indigenous governance partners are co-deliberating, and revisit decisions as technologies and contexts evolve. This iterative engagement strengthens accountability and supports ethically grounded innovation over time.

### Limitations

This structured narrative review has several limitations. Although a structured search strategy was employed and multiple databases and grey literature sources were consulted to capture a broad range of relevant publications, the narrative review methodology does not aim to identify all potentially eligible studies and therefore may be subject to selection bias. The defined scope and inclusion criteria may have resulted in the omission of some pertinent studies. In addition, although a subset of studies with uncertain eligibility was independently screened by the lead author and uncertainties or disagreements were resolved through discussion and consensus, the primary screening was conducted by a single reviewer. Consequently, an inter-reviewer agreement metric was not calculated, which may have increased the potential for selection bias. A single quality appraisal framework, Hawker et al.,^13^ was applied across the heterogeneous body of included evidence rather than using appraisal tools tailored to individual evidence types. While this provided a consistent approach to quality assessment, it may not have fully captured the methodological characteristics of all forms of evidence. The review focused specifically on literature addressing AI within digital health contexts involving Indigenous populations. The review was also limited to English-language publications and therefore may not have captured relevant scholarship from non-English-speaking regions, including Indigenous contexts in Latin America and the Pacific. Broader discussions of AI ethics or digital health governance that were not explicitly grounded in Indigenous contexts were excluded to preserve conceptual focus and relevance. For clarity and analytical coherence, ethical considerations were organized into six thematic domains. Some considerations, such as privacy and Indigenous data sovereignty, or co-design and accountability, were inherently interconnected and could reasonably span multiple categories. The thematic organization was intended to enhance the usability of the proposed framework and not to suggest rigid conceptual separation. The ethical domains should therefore be understood as interdependent and dynamically related. Finally, although the framework is grounded in published literature and informed by ongoing community-engaged research initiatives, it reflects themes identified within a limited evidence base and therefore remains conceptual and has not undergone formal empirical or technical validation. It does not claim to resolve all ethical tensions that may arise in AI-enabled digital health implementation. It was designed to support structured reflection, dialogue, and governance deliberation in evolving technological contexts. Future research should empirically evaluate, refine, and validate the framework through community-led implementation across diverse Indigenous contexts.

## Conclusion

AI is increasingly positioned as a solution to long-standing healthcare access challenges. Yet, without deliberate ethical governance, AI-enabled digital health initiatives risk reinforcing the very inequities they seek to address. For Indigenous communities in particular, technological innovation cannot be separated from experiences of data extraction, structural exclusion, and valid skepticism of health systems. Ethical integration must therefore move beyond compliance and toward relational accountability. This review identified six interrelated ethical domains that consistently shape responsible AI integration in Indigenous digital health contexts. These domains do not operate independently; they intersect through governance, trust, and shared authority over data and decision-making. The framework proposed in this paper does not offer predetermined answers. Instead, it creates structured space for critical questioning — asking who benefits, who decides, whose knowledge is recognized, and how harms are anticipated before they materialize. Importantly, ethical readiness should be understood as coequal to technological readiness. Infrastructure, algorithmic performance, and scalability are insufficient measures of success if community governance, cultural safety, and data sovereignty are not embedded from the outset. The integration of AI into Indigenous health systems requires a shift in orientation: from deploying tools into communities to developing systems with communities under shared leadership. As AI capabilities continue to evolve, ethical reflection must remain iterative, adaptive, and grounded in lived realities. The most durable digital health innovations will not be those that scale the fastest, but those that earn trust, build relationships, sustain partnerships, and redistribute decision-making authority. By centring governance, reciprocity, and accountability, AI-enabled digital health can move from being not only technologically advanced, but also ethically grounded and community-defined. The framework proposed in this review provides a conceptual foundation for ethical governance and establishes a basis for future empirical evaluation, technical validation, and continued refinement through partnerships with Indigenous communities. Through this ongoing process, AI-enabled digital health may more effectively contribute to narrowing persistent gaps in healthcare delivery for Indigenous populations.

## Supplemental material

Supplemental Material - Ethical governance of artificial intelligence in digital health for indigenous populations: A narrative review and conceptual framework Supplemental Material for Ethical governance of artificial intelligence in digital health for indigenous populations: A narrative review and conceptual framework by Amal Khan, Alyson S. N. Bear, Mairyn Rackow, Cassandra Opikokew Wajuntah, Kenneth Lai, Veronica McKinney, John Costa, Ivar Mendez in DIGITAL HEALTH.

Supplemental Material - Ethical governance of artificial intelligence in digital health for indigenous populations: A narrative review and conceptual framework Supplemental Material for Ethical governance of artificial intelligence in digital health for indigenous populations: A narrative review and conceptual framework by Amal Khan, Alyson S. N. Bear, Mairyn Rackow, Cassandra Opikokew Wajuntah, Kenneth Lai, Veronica McKinney, John Costa, Ivar Mendez in DIGITAL HEALTH.

Supplemental Material - Ethical governance of artificial intelligence in digital health for indigenous populations: A narrative review and conceptual framework Supplemental Material for Ethical governance of artificial intelligence in digital health for indigenous populations: A narrative review and conceptual framework by Amal Khan, Alyson S. N. Bear, Mairyn Rackow, Cassandra Opikokew Wajuntah, Kenneth Lai, Veronica McKinney, John Costa, Ivar Mendez in DIGITAL HEALTH.

Supplemental Material - Ethical governance of artificial intelligence in digital health for indigenous populations: A narrative review and conceptual framework Supplemental Material for Ethical governance of artificial intelligence in digital health for indigenous populations: A narrative review and conceptual framework by Amal Khan, Alyson S. N. Bear, Mairyn Rackow, Cassandra Opikokew Wajuntah, Kenneth Lai, Veronica McKinney, John Costa, Ivar Mendez in DIGITAL HEALTH.

Supplemental Material - Ethical governance of artificial intelligence in digital health for indigenous populations: A narrative review and conceptual framework Supplemental Material for Ethical governance of artificial intelligence in digital health for indigenous populations: A narrative review and conceptual framework by Amal Khan, Alyson S. N. Bear, Mairyn Rackow, Cassandra Opikokew Wajuntah, Kenneth Lai, Veronica McKinney, John Costa, Ivar Mendez in DIGITAL HEALTH.
