# Supplementary material for: Ethical governance of artificial intelligence in digital health for Indigenous populations: A narrative review and conceptual framework
Source: Digit Health. 2026 Aug 22;12:20552076261481276. doi: 10.1177/20552076261481276 (PMC13500953; doi:10.1177/20552076261481276)
Supplement: Supplementary file 1 [file sj-pdf-1-dhj-10.1177_20552076261481276.pdf]

Search strategy worksheet

Search statement or topic

Indicate your search topic or research question.

What regulations are in place in Canada or have been suggested in published Canadian literature to ensure ethical use of AI for Indigenous populations in virtual healthcare systems?

Major concepts

Identify the main concepts of your topic.

1.	2.	3.	4.	5.
Regulations	Ethical use	AI	Suggested	Indigenous

Search strategy

Use the main concepts and translate these into keywords and synonyms (don't forget about **" "** and *****).

	**Concept 1**	**AND Concept 2**	**AND Concept 3**	**AND Concept 4**	**AND Concept 5**
	Regulat*	Ethic*	"artificial intelligence"	Suggest*	Indig*
**OR**	Rule	Moral*	"Machine learning"	Recommend*	Aborig*
**OR**	Act*	Social	"Deep learning"	Propos*	First Nation*
**OR**	Law*	Equit*	"knowledge engineering"	Submit*	Inuit
**OR**	Guidance	"data sovereignty"	"Neural network"	Advice	Metis/ Métis
**OR**	Directive*	"privacy protection"	"Intelligent retrieval"	Advis*	Native*
**OR**	Report*	"responsible use"	Robot*	Guid*	Indian*
**OR**	Guideline*	"diversity, equity, inclusion"	Digital health	"best practice"	
**OR**	Jurisprudence		Telemedicine		
**OR**			"intelligent system"		
			"generative artificial intelligence"		

Source: Rielding, A.M. (2002). *Learning to Learn*. New York NY: Neal-Schuman Publishers.
