# Supplementary material for: Ethical governance of artificial intelligence in digital health for Indigenous populations: A narrative review and conceptual framework
Source: Digit Health. 2026 Aug 22;12:20552076261481276. doi: 10.1177/20552076261481276 (PMC13500953; doi:10.1177/20552076261481276)
Supplement: Supplementary file 2 [file sj-pdf-2-dhj-10.1177_20552076261481276.pdf]

Database(s): **Ovid MEDLINE(R) ALL** 1946 to July 25, 2025
Search Strategy:

#	Searches	Results
1	exp Artificial Intelligence/ or (AI or "artificial intelligence" or AIVI or "classification algorithm*" or "computer heuristic*" or "convolutional network*" or DALL-E or "decision support system*" or "decision tree" or DeepAI or "deep learning" or "data science" or "feature detection" or "generative pre-trained transformer" or "generative pretrained transformer" or Invideo or "language learning model*" or "large language model*" or "learning algorithm*" or "machine learning" or (Markov adj3 model*) or Midjourney or ((multifactor* or multicriteria) adj3 ("decision analysis" or "decision making")) or "natural language process*" or "nearest neighbo*" or "neural network*" or "outlier detection" or "pattern recognition" or Perplexity or "probability tree" or "random forest" or "representation learning" or Runway AI or Runway Gen-1 or "Stable Diffusion" or "support vector machine*" or "transfer learning" or "Bing chat" or ChatGPT* or "Chat GPT" or "Google* Bard" or "Google* Gemini" or "IBM Watson" or "Microsoft* Bing" or "Microsoft* Copilot" or OpenAI or "Open AI" or PathAI or "Path AI" or DeepSeek or Grok).mp.[1]	643839
2	((("artificial intelligence" or AI) adj2 (chat* or generat*)) or GenAI or ((large or natural or generative or machine or deep learning) adj3 (language or text) adj3 model*) or AlexaTM or (Amazon* and Alexa) or Anthropic or Bard or Bardeen or BERT or "Bing chat" or BioGPT or BLOOM or BloombergGPT or Cerebras-GPT or ChatGPT* or "Chat GPT" or chatbot* or Chatsonic or Chinchilla or Claude or DALL-E or EinsteinGPT or Ernie or Falcon or Galactica or "Generative Fill" or "GitHub Copilot" or GLaM or "Google* Assistant" or "Google* Bard" or "Google* Gemini" or Gopher or GPT-1 or GPT-2 or GPT-3* or GPT-4* or GPTNeo or GPT-NEoX or GPT-J* or "IBM Watson" or LaMDA or LLaMA or "Megatron-Turing NLG" or "Microsoft* Bing" or "Microsoft* Copilot" or Midjourney or Minerva or NeevaAI or Nvidia or OpenAI or "Open AI" or OpenAssistant or PaLM or PanGu-E or PathAI or "Path AI" or Perplexity or "pre-trained transformer*" or "pretrained transformer*" or (Apple* and Siri) or SlackGPT or "Stable Diffusion" or StyleGAN or Synthesia or XLNet or YaLM 100B or YouChat or DeepSeek or Grok or Elicit or Perplexity).mp.[2]	150323
3	1 or 2	772657
4	exp Telemedicine/ or exp Remote Consultation/ or (cyberthera* or telecare or telecollaborat* or teleconsult* or teleconference* or teleeducat* or telediagnos* or telehealth or teleguide* or telediagnos* or telelearn* or telemed* or telementor* or telemonitor* or teleneurol* or teleopth* or telepediatric* or telepresence* or telerehab* or telerobotic* or telescreen* or teletherap* or teletransmi* or mhealth or "m heath" or ehealth* or "e health" or ((cyber or digital or remote* or distance* or tele) adj2 (care or collaborat* or consult* or conference* or educat* or diagnos* or health or guide* or diagnos* or learn* or med* or mentor* or monitor* or	198635

	presence* or screen* or therap* or transmi*)) or ((cyber or digital or distance* or tele or remote* or sms or phone* or internet or "web based" or telephone* or texting or "mobile app*" or Instagram or Snapchat or Facetime or GMeet* or hangout* or Skype or Zoom or Web-ex or WebEx or Bluejeans or Facebook or e-mail* or email* or "e chat" or echat or "social media" or "text message*" or "answering machine*" or "voice mail*" or "video conferenc*" or "video link*" or "video chat*") adj3 (consult* or support* or diagnos* or "follow-up*" or "health" or doctor* or "primary care" or clinic or clinics or clinician* or nurs* or psycholog* or therap* or intervention* or delivery))).mp.[3]	
5	exp Ethics/ or exp Privacy/ or exp Morals/ or Paternalism/ or exp Prejudice/ or Social Values/ or Stereotyping/ or Social Stigma/ or Medically Underserved Area/ or exp Health Services Accessibility/ or Healthcare Disparities/ or exp Disclosure/ or exp Human Rights/ or exp Social Problems/ or (ethic* or privacy or moral* or paternal* or prejudic* or social value* or stereotyp* or "health service* access*" or "healthcare disparit*" or disclosure or "human right*" or "social problem*" or ((healthcare or "health care" or nonclinical or "non clinical" or "community based" or "public health" or "preventive care") adj3 (access* or deliver* or distribut* or system*)) or bioethics* or stigma* or equal* or inequal* or unequal* or equit* or inequit* or disparit* or fair* or unfair* or (social adj (responsib* or obligat* or justice)) or ((social or psychosocial or psychological) adj2 (impact* or burden*)) or harm* or confidential* or "information provision" or ((conflict or financial or indust*) adj3 interest*) or (industry adj3 (funding or involvement or sponsor*)) or autonomy* or transparen*).mp.[4]	2534321
6	cultural diversity/ or diversity, equity, inclusion/ or indigenous peoples/ or "american indian or alaska native"/ or indians, central american/ or indians, north american/ or alaska natives/ or indigenous canadians/ or inuit/ or navajo people/ or pima people/ or indians, south american/ or "australian aboriginal and torres strait islander peoples"/ or maori people/ or "native hawaiian or pacific islander"/ or (indigenous* or inuit* or maori or "American Indian*" or navajo or pima* or "pacific islander*" or metis* or aborigin* or native* or Amerind* or autochton* or "first nation*" or innu or inuk or Inuvialuit or trib* or Australoid or "torres strait*" or "first australian" or koorie or koori or murri or tiwi or nyoongar or noongar or nyoongah or nyungar or goori or Yolngu or anangu or palawa or nunga or ngunnawal or ngunawal or amata or blackstone or papulankutja or djalkiripuynu or bardi or jawi or bwgcolman or warrwa or "akimel oodham" or anishinabek or anishnabek or anishnawbe or anishinaabe or anishinaabeg or aleut* or algonquin* or algonkian* or algonquian* or arikara* or sahnish or athabasca* or athabaskan* or athapascan* or athapaskan* or apache* or caddoan* or chipewyan or chippewa or cree or cherokee* or choctaw* or dene or eskimo* or gitskan or haudenosaunee or haida* or hopi or hopis or huron or huron-wendat or inuuk or inupiat* or inupiaq or iroquois or iroquoian or kwakiutl or lakota or miawpukek or micmac or mikmaq or mi'kmaq or mohawk* or mushkegowuk or muscogee or muskogee or muskogean or nisgaa or nisga'a or nakoda or nakota or navaho or naabeeho or ojibway or opaskwayak or ojibwe or ojibwa or "ouje bougoumou" or papago* or "tohono o'odham" or "tohono oodham" or pauktuutit or qikiqtani or sioux or siouan or tlingit* or unangan or "vuntut gwitchin" or yaqui* or yupik or kanaka or (American adj2 samoa) or "original inhabitant*" or autochthon* or "first people*" or band* or chief*	1017899

	or on-reserve or off-reserve or reserve or "non-status Indian" or "nonstatus Indian" or "non status Indian" or "treaty Indian" or Saulteaux or Ktunaxa or Assiniboine or Naskapi or oji-cree or anishininew or Tsimshian or gitsxan or Haisla or Heiltsuk or kwakwaka'wakw or "nuu chah nulth" or nuu-chah-nulth or tsilhqot'in or Dakelh or wet'suwet'en or sekani or tse'khene or tsekhene or dunne-za or dane-zaa or Tahltan or nahani or kaska or "kaska dena" or Tagish or Tutchone or nuxalk or salish or Okanagan or Syilx or Suknaqinx or Okinagan or Secwepemc or Shuswap or blackfoot or tsuut'ina or Sarcee or sarsi or gwich'in or han or potawatomi or pottawatomi or pottawatomie or Maliseet or Passamaquoddy or cayuga or Oneida or onondaga or Seneca or Tuscarora or Wyandot or "red road").mp.	
7	((marginali* or underserve* or raciali* or vulnerable or rural or traditional or "racial minority") adj3 (population* or person* or people* or individual* or woman or women or female* or male* or man or men or adult* or famil* or youth* or adolescen* or teen* or child* or boy* or girl* or communit* or group* or nation* or trib* or band* or reserve* or village*)).mp. [mp=title, book title, abstract, original title, name of substance word, subject heading word, floating sub-heading word, keyword heading word, organism supplementary concept word, protocol supplementary concept word, rare disease supplementary concept word, unique identifier, synonyms, population supplementary concept word, anatomy supplementary concept word]	219342
8	6 or 7	1224020
9	united states/ or appalachian region/ or alabama/ or georgia/ or kentucky/ or maryland/ or new york/ or north carolina/ or ohio/ or pennsylvania/ or south carolina/ or tennessee/ or virginia/ or west virginia/ or great lakes region/ or illinois/ or chicago/ or indiana/ or michigan/ or minnesota/ or new york city/ or wisconsin/ or mid-atlantic region/ or delaware/ or "district of columbia"/ or baltimore/ or new jersey/ or philadelphia/ or midwestern united states/ or iowa/ or kansas/ or missouri/ or nebraska/ or north dakota/ or oklahoma/ or south dakota/ or new england/ or connecticut/ or maine/ or massachusetts/ or boston/ or new hampshire/ or rhode island/ or vermont/ or northwestern united states/ or idaho/ or montana/ or oregon/ or washington/ or wyoming/ or pacific states/ or alaska/ or california/ or los angeles/ or san francisco/ or hawaii/ or southeastern united states/ or arkansas/ or florida/ or louisiana/ or new orleans/ or mississippi/ or southwestern united states/ or arizona/ or colorado/ or nevada/ or new mexico/ or texas/ or utah/ or ("united states" or USA or "appalachian region" or alabama or georgia or kentucky or maryland or "new york" or "north carolina" or ohio or pennsylvania or "south carolina" or tennessee or virginia or "west virginia" or "great lakes region" or illinois or chicago or indiana or michigan or minnesota or "new york city" or NYC or Wisconsin or "mid-atlantic region" or "mid Atlantic region" or delaware or "district of columbia" or baltimore or "new jersey" or Philadelphia or iowa or kansas or missouri or nebraska or "north dakota" or oklahoma or "south dakota" or "new england" or connecticut or maine or massachusetts or boston or "new hampshire" or "rhode island" or Vermont or idaho or montana or oregon or washington or Wyoming or "pacific states" or Alaska or California or "los angeles" or "san Francisco" or Hawaii or Arkansas or florida or louisiana or "new orleans" or Mississippi or Arizona or	2373485

	Colorado or Nevada or "new mexico" or texas or Utah or American*).mp.	
10	canada/ or alberta/ or british columbia/ or manitoba/ or new brunswick/ or "newfoundland and labrador"/ or northwest territories/ or nova scotia/ or nunavut/ or ontario/ or prince edward island/ or quebec/ or saskatchewan/ or yukon territory/ or ("North America" or Canada or Alberta or "British Columbia" or Manitoba or "new Brunswick" or "newfoundland and labrador" or "northwest territories" or "nova scotia" or Nunavut or Ontario or "prince Edward island" or Quebec or Saskatchewan or "Yukon territory" or Canadian*).mp.	353687
11	united kingdom/ or channel islands/ or guernsey/ or england/ or london/ or northern ireland/ or scotland/ or hebrides/ or wales/ or ("united kingdom" or UK or "channel islands" or guernsey or England or London or Ireland or Scotland or Hebrides or wales or English or British or Scottish or Irish or Welsh or Hebridean*).mp.	2419850
12	australia/ or australian capital territory/ or new south wales/ or northern territory/ or queensland/ or south australia/ or tasmania/ or victoria/ or western australia/ or (Australia or "new south wales" or "northern territory" or queensland or Tasmania or Victoria or Australian*).mp.	267098
13	new zealand/ or australasia/ or ("new zealand" or Australasia or Aotearoa or "new Zealander*").mp.	91337
14	9 or 10 or 11 or 12 or 13	5190215
15	3 and 4 and 5 and 8 and 14	140
16	limit 15 to (english language and yr="2020 - 2025")	108

Database(s): **Web of Science**
Search strategy:

#	Searches	Results
1	AI OR "artificial intelligence" OR AIVI OR "classification algorithm*" OR "computer heuristic*" OR "convolutional network*" OR DALL-E OR "decision support system*" OR "decision tree" OR DeepAI OR "deep learning" OR "data science" OR "feature detection" OR "generative pre-trained transformer" OR "generative pretrained transformer" OR Invideo OR "language learning model*" OR "large language model*" OR "learning algorithm*" OR "machine learning" OR (Markov NEAR/3 model*) OR Midjourney OR ((multifactor* OR multicriteria) NEAR/3 ("decision analysis" OR "decision making")) OR "natural language	2178945

	process*" OR "nearest neighbo*" OR "neural network*" OR "outlier detection" OR "pattern recognition" OR Perplexity OR "probability tree" OR "random forest" OR "representation learning" OR "Runway AI" OR "Runway Gen-1" OR "Stable Diffusion" OR "support vector machine*" OR "transfer learning" OR "Bing chat" OR ChatGPT* OR "Chat GPT" OR "Google* Bard" OR "Google* Gemini" OR "IBM Watson" OR "Microsoft* Bing" OR "Microsoft* Copilot" OR OpenAI OR "Open AI" OR PathAI OR "Path AI" OR DeepSeek OR Grok (Topic)1	
2	(("artificial intelligence" or AI) adj2 (chat* or generat*)) or GenAI or ((large or natural or generative or machine or deep learning) adj3 (language or text) adj3 model*) or AlexaTM or (Amazon* and Alexa) or Anthropic or Bard or Bardeen or BERT or "Bing chat" or BioGPT or BLOOM or BloombergGPT or Cerebras-GPT or ChatGPT* or "Chat GPT" or chatbot* or Chatsonic or Chinchilla or Claude or DALL-E or EinsteinGPT or Ernie or Falcon or Galactica or "Generative Fill" or "GitHub Copilot" or GLaM or "Google* Assistant" or "Google* Bard" or "Google* Gemini" or Gopher or GPT-1 or GPT-2 or GPT-3* or GPT-4* or GPTNeo or GPT-NEoX or GPT-J* or "IBM Watson" or LaMDA or LLaMA or "Megatron-Turing NLG" or "Microsoft* Bing" or "Microsoft* Copilot" or Midjourney or Minerva or NeevaAI or Nvidia or OpenAI or "Open AI" or OpenAssistant or PaLM or PanGu-E or PathAI or "Path AI" or Perplexity or "pre-trained transformer*" or "pretrained transformer*" or (Apple* and Siri) or SlackGPT or "Stable Diffusion" or StyleGAN or Synthesia or XLNet or YaLM 100B or YouChat or DeepSeek or Grok or Elicit or Perplexity (Topic)[2]	602031
3	#1 OR #2	2725014
4	cyberthera* OR telecare OR telecollaborat* OR teleconsult* OR teleconference* OR teleeducat* OR telediagnos* OR telehealth OR teleguide* OR telediagnos* OR telelearn* OR telemed* OR telementor* OR telemonitor* OR teleneurol* OR teleopth* OR telepediatric* OR telepresence* OR telerehab* OR telerobotic* OR telescreen* OR teletherap* OR teletransmi* OR mhealth OR "m heath" OR ehealth* OR "e health" OR ((cyber OR digital OR remote* OR distance* OR tele) NEAR/2 (care OR collaborat* OR consult* OR conference* OR educat* OR diagnos* OR health OR guide* OR diagnos* OR learn* OR med* OR mentor* OR monitor* OR presence* OR screen* OR therap* OR transmi*)) OR ((cyber OR digital OR distance* OR tele OR remote* OR sms OR phone* OR internet OR "web based" OR telephone* OR texting OR "mobile app*" OR Instagram OR Snapchat OR Facetime OR GMeet* OR hangout* OR Skype OR Zoom OR Web-ex OR WebEx OR Bluejeans OR Facebook OR e-mail* OR email* OR "e chat" OR echat OR "social media" OR "text message*" OR "answering machine*" OR "voice mail*" OR "video conferenc*" OR "video link*" OR "video chat*") NEAR/3 (consult* OR support* OR diagnos* OR follow-up* OR health OR doctor* OR "primary care" OR clinic OR clinics OR clinician* OR nurs* OR psycholog* OR therap* OR intervention* OR delivery)) (Topic)[3]	409124
5	ethic* OR privacy OR moral* OR paternal* OR prejudic* OR "social value*" OR stereotyp* OR "health service* access*" OR	5139981

	"healthcare disparit*" OR disclosure OR "human right*" OR "social problem*" OR ((healthcare OR "health care" OR nonclinical OR "non clinical" OR "community based" OR "public health" OR "preventive care") NEAR/3 (access* OR deliver* OR distribut* OR system*)) OR bioethics* OR stigma* OR equal* OR inequal* OR unequal* OR equit* OR inequit* OR disparit* OR fair* OR unfair* OR (social NEAR/0 (responsib* OR obligat* OR justice)) OR ((social OR psychosocial OR psychological) NEAR/2 (impact* OR burden*)) OR harm* OR confidential* OR "information provision" OR ((conflict OR financial OR indust*) NEAR/3 interest*) OR (industry NEAR/3 (funding OR involvement OR sponsor*)) OR autonomy* OR transparen* (Topic)[4]	
6	indigenous* OR inuit* OR maori OR "American Indian*" OR navajo OR pima* OR "pacific islander*" OR metis* OR aborigin* OR native* OR Amerind* OR autochton* OR "first nation*" OR innu OR inuk OR Inuvialuit OR trib* OR Australoid OR "torres strait*" OR "first australian" OR koorie OR koori OR murri OR tiwi OR nyoongar OR noongar OR nyoongah OR nyungar OR goori OR Yolngu OR anangu OR palawa OR nunga OR ngunnawal OR ngunawal OR amata OR blackstone OR papulankutja OR djalkiripuynu OR bardi OR jawi OR bwgcolman OR warrwa OR "akimel oodham" OR anishinabek OR anishnabek OR anishnawbe OR anishinaabe OR anishinaabeg OR aleut* OR algonquin* OR algonkian* OR algonquian* OR arikara* OR sahnish OR athabasca* OR athabaskan* OR athapascan* OR athapaskan* OR apache* OR caddoan* OR chipewyan OR chippewa OR cree OR cherokee* OR choctaw* OR dene OR eskimo* OR gitskan OR haudenosaunee OR haida* OR hopi OR hopis OR huron OR huron-wendat OR inuuk OR inupiat* OR inupiaq OR iroquois OR iroquoian OR kwakiutl OR lakota OR miawpukek OR micmac OR mikmaq OR mi'kmaq OR mohawk* OR mushkegowuk OR muscogee OR muskogee OR muskogean OR nisgaa OR nisga'a OR nakoda OR nakota OR navaho OR naabeeho OR ojibway OR opaskwayak OR ojibwe OR ojibwa OR "ouje bougoumou" OR papago* OR "tohono o'odham" OR "tohono oodham" OR pauktuutit OR qikiqtani OR sioux OR siouan OR tlingit* OR unangan OR "vuntut gwitchin" OR yaqui* OR yupik OR kanaka OR (American NEAR/2 samoa) OR "original inhabitant*" OR autochthon* OR "first people*" OR band* OR chief* OR on-reserve OR off-reserve OR reserve OR "non-status Indian" OR "nonstatus Indian" OR "non status Indian" OR "treaty Indian" OR Saulteaux OR Ktunaxa OR Assiniboine OR Naskapi OR oji-cree OR anishininew OR Tsimshian OR gitsxan OR Haisla OR Heiltsuk OR kwakwaka'wakw OR "nuu chah nulth" OR nuu-chah-nulth OR tsilhqot'in OR Dakelh OR wet'suwet'en OR sekani OR tse'khene OR tsekhene OR dunne-za OR dane-zaa OR Tahltan OR nahani OR kaska OR "kaska dena" OR Tagish OR Tutchone OR nuxalk OR salish OR Okanagan OR Syilx OR Suknaqinx OR Okinagan OR Secwepemc OR Shuswap OR blackfoot OR tsuut'ina OR Sarcee OR sarsi OR gwich'in OR han OR potawatomi OR pottawatomi OR pottawatomie OR Maliseet OR Passamaquoddy OR cayuga OR Oneida OR onondaga OR Seneca OR Tuscarora OR Wyandot OR "red road" (Topic)	8211967
7	(marginali* OR underserve* OR raciali* OR vulnerable OR rural OR traditional OR "racial minority") NEAR/3 (population* OR person* OR people* OR individual* OR woman OR women OR female* OR male* OR man OR men OR adult* OR famil* OR youth* OR adolescen* OR teen* OR child* OR boy* OR girl* OR communit* OR group* OR nation* OR trib* OR band* OR	312555

	reserve* OR village*) (Topic)	
8	#6 OR #7	8485042
9	"united states" or USA or "appalachian region" or alabama or georgia or kentucky or maryland or "new york" or "north carolina" or ohio or pennsylvania or "south carolina" or tennessee or virginia or "west virginia" or "great lakes region" or illinois or chicago or indiana or michigan or minnesota or "new york city" or NYC or Wisconsin or "mid-atlantic region" or "mid Atlantic region" or delaware or "district of columbia" or baltimore or "new jersey" or Philadelphia or iowa or kansas or missouri or nebraska or "north dakota" or oklahoma or "south dakota" or "new england" or connecticut or maine or massachusetts or boston or "new hampshire" or "rhode island" or Vermont or idaho or montana or oregon or washington or Wyoming or "pacific states" or Alaska or California or "los angeles" or "san Francisco" or Hawaii or Arkansas or florida or louisiana or "new orleans" or Mississippi or Arizona or Colorado or Nevada or "new mexico" or texas or Utah or American* (Topic)	3822741
10	"North America" or Canada or Alberta or "British Columbia" or Manitoba or "new Brunswick" or "newfoundland and labrador" or "northwest territories" or "nova scotia" or Nunavut or Ontario or "prince Edward island" or Quebec or Saskatchewan or "Yukon territory" or Canadian* (Topic)	694817
11	"united kingdom" or UK or "channel islands" or guernsey or England or London or Ireland or Scotland or Hebrides or wales or English or British or Scottish or Irish or Welsh or Hebridean* (Topic)	1768556
12	Australia or "new south wales" or "northern territory" or queensland or Tasmania or Victoria or Australian* (Topic)	576606
13	"new zealand" or Australasia or Aotearoa or "new Zealander*" (Topic)	168864
14	#9 OR #10 OR #11 OR #12 OR #13	6479833
15	#3 AND #4 AND #5 AND #8 AND #14 and 2020 or 2021 or 2022 or 2023 or 2024 or 2025 (Publication Years) and English (Languages)	163

Database(s): **Scopus**
Search strategy:

#	Searches	Results

1	((TITLE-ABS-KEY (AI OR "artificial intelligence" OR AIVI OR "classification algorithm*" OR "computer heuristic*" OR "convolutional network*" OR DALL-E OR "decision support system*" OR "decision tree" OR DeepAI OR "deep learning" OR "data science" OR "feature detection" OR "generative pre-trained transformer" OR "generative pretrained transformer" OR Invideo OR "language learning model*" OR "large language model*" OR "learning algorithm*" OR "machine learning" OR (Markov W/3 model*) OR Midjourney OR ((multifactor* OR multicriteria) W/3 ("decision analysis" OR "decision making")) OR "natural language process*" OR "nearest neighbo*" OR "neural network*" OR "outlier detection" OR "pattern recognition" OR Perplexity OR "probability tree" OR "random forest" OR "representation learning" OR "Runway AI" OR "Runway Gen-1" OR "Stable Diffusion" OR "support vector machine*" OR "transfer learning" OR "Bing chat" OR ChatGPT* OR "Chat GPT" OR "Google* Bard" OR "Google* Gemini" OR "IBM Watson" OR "Microsoft* Bing" OR "Microsoft* Copilot" OR OpenAI OR "Open AI" OR PathAI OR "Path AI" OR DeepSeek OR Grok))[1] OR (TITLE-ABS-KEY ((("artificial intelligence" OR AI) W/2 (chat* OR generat*)) OR GenAI OR ((large OR natural OR generative OR machine OR "deep learning") W/3 (language OR text) W/3 model*) OR AlexaTM OR (Amazon* AND Alexa) OR Anthropic OR Bard OR Bardeen OR BERT OR "Bing chat" OR BioGPT OR BLOOM OR BloombergGPT OR Cerebras-GPT OR ChatGPT* OR "Chat GPT" OR chatbot* OR Chatsonic OR Chinchilla OR Claude OR DALL-E OR EinsteinGPT OR Ernie OR Falcon OR Galactica OR "Generative Fill" OR "GitHub Copilot" OR GLaM OR "Google* Assistant" OR "Google* Bard" OR "Google* Gemini" OR Gopher OR GPT-1 OR GPT-2 OR GPT-3* OR GPT-4* OR GPTNeo OR GPT-NEoX OR GPT-J* OR "IBM Watson" OR LaMDA OR LLaMA OR "Megatron-Turing NLG" OR "Microsoft* Bing" OR "Microsoft* Copilot" OR Midjourney OR Minerva OR NeevaAI OR Nvidia OR OpenAI OR "Open AI" OR OpenAssistant OR PaLM OR PanGu-E OR PathAI OR "Path AI" OR Perplexity OR "pre-trained transformer*" OR "pretrained transformer*" OR (Apple* AND Siri) OR SlackGPT OR "Stable Diffusion" OR StyleGAN OR Synthesia OR XLNet OR "YaLM 100B" OR YouChat OR DeepSeek OR Grok OR Elicit OR Perplexity)))[2] AND (TITLE-ABS-KEY (cyberthera* OR telecare OR telecollaborat* OR teleconsult* OR teleconference* OR teleeducat* OR telediagnos* OR telehealth OR teleguide* OR telediagnos* OR telelearn* OR telemed* OR telementor* OR telemonitor* OR teleneurol* OR teleopth* OR telepediatric* OR telepresence* OR telerehab* OR telerobotic* OR telescreen* OR teletherap* OR teletransmi* OR mhealth OR "m heath" OR ehealth* OR "e health" OR ((cyber OR digital OR remote* OR distance* OR tele) W/2 (care OR collaborat* OR consult* OR conference* OR educat* OR diagnos* OR health OR guide* OR diagnos* OR learn* OR med* OR mentor* OR monitor* OR presence* OR screen* OR therap* OR transmi*)) OR ((cyber OR digital OR distance* OR tele OR remote* OR sms OR phone* OR internet OR "web based" OR telephone* OR texting OR "mobile app*" OR Instagram OR Snapchat OR Facetime OR GMeet* OR hangout* OR Skype OR Zoom OR Web-ex OR WebEx OR Bluejeans OR Facebook OR e-mail* OR email* OR "e chat" OR echat OR "social media" OR "text message*" OR "answering machine*" OR "voice mail*" OR "video conferenc*" OR "video link*" OR "video chat*") W/3 (consult* OR support* OR diagnos* OR follow-up* OR health OR doctor* OR "primary care" OR clinic OR clinics OR clinician* OR nurs* OR psycholog* OR therap* OR intervention* OR delivery))))[3] AND (TITLE-ABS-KEY (ethic* OR privacy OR moral* OR paternal* OR	169

	prejudic* OR "social value*" OR stereotyp* OR "health service* access*" OR "healthcare disparit*" OR disclosure OR "human right*" OR "social problem*" OR ((healthcare OR "health care" OR nonclinical OR "non clinical" OR "community based" OR "public health" OR "preventive care") W/3 (access* OR deliver* OR distribut* OR system*)) OR bioethics* OR stigma* OR equal* OR inequal* OR unequal* OR equit* OR inequit* OR disparit* OR fair* OR unfair* OR (social W/1 (responsib* OR obligat* OR justice)) OR ((social OR psychosocial OR psychological) W/2 (impact* OR burden*)) OR harm* OR confidential* OR "information provision" OR ((conflict OR financial OR indust*) W/3 interest*) OR (industry W/3 (funding OR involvement OR sponsor*)) OR autonomy* OR transparen*))[4] AND ((TITLE-ABS-KEY (indigenous* OR inuit* OR maori OR "American Indian*" OR navajo OR pima* OR "pacific islander*" OR metis* OR aborigin* OR native* OR Amerind* OR autochton* OR "first nation*" OR innu OR inuk OR Inuvialuit OR trib* OR Australoid OR "torres strait*" OR "first australian" OR koorie OR koori OR murri OR tiwi OR nyoongar OR noongar OR nyoongah OR nyungar OR goori OR Yolngu OR anangu OR palawa OR nunga OR ngunnawal OR ngunawal OR amata OR blackstone OR papulankutja OR djalkiripuynu OR bardi OR jawi OR bwgcolman OR warrwa OR "akimel oodham" OR anishinabek OR anishnabek OR anishnawbe OR anishinaabe OR anishinaabeg OR aleut* OR algonquin* OR algonkian* OR algonquian* OR arikara* OR sahnish OR athabasca* OR athabaskan* OR athapascan* OR athapaskan* OR apache* OR caddoan* OR chipewyan OR chippewa OR cree OR cherokee* OR choctaw* OR dene OR eskimo* OR gitskan OR haudenosaunee OR haida* OR hopi OR hopis OR huron OR huron-wendat OR inuuk OR inupiat* OR inupiaq OR iroquois OR iroquoian OR kwakiutl OR lakota OR miawpukek OR micmac OR mikmaq OR mi'kmaq OR mohawk* OR mushkegowuk OR muscogee OR muskogee OR muskogean OR nisgaa OR nisga'a OR nakoda OR nakota OR navaho OR naabeeho OR ojibway OR opaskwayak OR ojibwe OR ojibwa OR "ouje bougoumou" OR papago* OR "tohono o'odham" OR "tohono oodham" OR pauktuutit OR qikiqtani OR sioux OR siouan OR tlingit* OR unangan OR "vuntut gwitchin" OR yaqui* OR yupik OR kanaka OR (American W/2 samoa) OR "original inhabitant*" OR autochthon* OR "first people*" OR band* OR chief* OR on-reserve OR off-reserve OR reserve OR "non-status Indian" OR "nonstatus Indian" OR "non status Indian" OR "treaty Indian" OR Saulteaux OR Ktunaxa OR Assiniboine OR Naskapi OR oji-cree OR anishininew OR Tsimshian OR gitsxan OR Haisla OR Heiltsuk OR kwakwaka'wakw OR "nuu chah nulth" OR nuu-chah-nulth OR tsilhqot'in OR Dakelh OR wet'suwet'en OR sekani OR tse'khene OR tsekhene OR dunne-za OR dane-zaa OR Tahltan OR nahani OR kaska OR "kaska dena" OR Tagish OR Tutchone OR nuxalk OR salish OR Okanagan OR Syilx OR Suknaqinx OR Okinagan OR Secwepemc OR Shuswap OR blackfoot OR tsuut'ina OR Sarcee OR sarsi OR gwich'in OR han OR potawatomi OR pottawatomi OR pottawatomie OR Maliseet OR Passamaquoddy OR cayuga OR Oneida OR onondaga OR Seneca OR Tuscarora OR Wyandot OR "red road")) OR (TITLE-ABS-KEY ((marginali* OR underserve* OR raciali* OR vulnerable OR rural OR traditional OR "racial minority") W/3 (population* OR person* OR people* OR individual* OR woman OR women OR female* OR male* OR man OR men OR adult* OR famil* OR youth* OR adolescen* OR teen* OR child* OR boy* OR girl* OR communit* OR group* OR nation* OR trib* OR band* OR reserve* OR village*))))) AND ((TITLE-ABS-KEY ("united states" or USA or "appalachian region" or alabama or georgia or kentucky or maryland or "new	

	york" or "north carolina" or ohio or pennsylvania or "south carolina" or tennessee or virginia or "west virginia" or "great lakes region" or illinois or chicago or indiana or michigan or minnesota or "new york city" or NYC or Wisconsin or "mid-atlantic region" or "mid Atlantic region" or delaware or "district of columbia" or baltimore or "new jersey" or Philadelphia or iowa or kansas or missouri or nebraska or "north dakota" or oklahoma or "south dakota" or "new england" or connecticut or maine or massachusetts or boston or "new hampshire" or "rhode island" or Vermont or idaho or montana or oregon or washington or Wyoming or "pacific states" or Alaska or California or "los angeles" or "san Francisco" or Hawaii or Arkansas or florida or louisiana or "new orleans" or Mississippi or Arizona or Colorado or Nevada or "new mexico" or texas or Utah or American*)) OR (TITLE-ABS-KEY ("North America" or Canada or Alberta or "British Columbia" or Manitoba or "new Brunswick" or "newfoundland and labrador" or "northwest territories" or "nova scotia" or Nunavut or Ontario or "prince Edward island" or Quebec or Saskatchewan or "Yukon territory" or Canadian*)) OR (TITLE-ABS-KEY ("united kingdom" or UK or "channel islands" or guernsey or England or London or Ireland or Scotland or Hebrides or wales or English or British or Scottish or Irish or Welsh or Hebridean*)) OR (TITLE-ABS-KEY (Australia or "new south wales" or "northern territory" or queensland or Tasmania or Victoria or Australian*)) OR (TITLE-ABS-KEY ("new zealand" or Australasia or Aotearoa or "new Zealander*"))) AND PUBYEAR > 2019 AND PUBYEAR < 2026 AND (LIMIT-TO (LANGUAGE,"English"))	

1. Campbell SM, Kung J. Filter to Retrieve Studies Related to Artificial Intelligence from the OVID MEDLINE Database. Geoffrey & Robyn Sperber Health Sciences Library, University of Alberta. Rev June 11, 2025. https://docs.google.com/document/d/1eWyO0jv9_6FYsxyC5LUYwFe9eH_3h83-tPNZ6wmos18/edit#heading=h.qi55eeyvgzy9
2. Kung JY & Chojecki D. Filter to Retrieve Studies Related to Generative AI from the OVID MEDLINE Database. Geoffrey & Robyn Sperber Health Sciences Library, University of Alberta. Rev May 3, 2025. https://docs.google.com/document/d/1fPiY7GmI_Z4xZQTqZ58DySUTJousLlZr1GTCP-kDOLA/edit#heading=h.qi55eeyvgzy9
3. Campbell, SM. Filter to Retrieve Studies Related to Remote Consultation and Care from the OVID Medline Database. Geoffrey & Robyn Sperber Health Sciences Library, University of Alberta. Rev. August 9, 2021. https://docs.google.com/document/d/1X2J_XZuIEOmtWCEvXEDFiCvtipGszTux_wV8c1gDHd4/edit#heading=h.qi55eeyvgzy9
4. Adapted from:
Ethics, Legal and Social Issues (ELSI) - MEDLINE. In: Canada's Drug Agency Search Filters Database. Ottawa: Canada's Drug Agency; 2025: https://searchfilters.cda-amc.ca/link/22. Accessed 2025-08-12.
