# Supplementary material for: Ethical governance of artificial intelligence in digital health for Indigenous populations: A narrative review and conceptual framework
Source: Digit Health. 2026 Aug 22;12:20552076261481276. doi: 10.1177/20552076261481276 (PMC13500953; doi:10.1177/20552076261481276)
Supplement: Supplementary file 3 [file sj-pdf-3-dhj-10.1177_20552076261481276.pdf]

# Grey Literature Search

(as adapted from: University of Toronto Libraries. Searching the Literature: A Guide to Comprehensive Searching in the Health Sciences [online]. 2025 Jul 25. Available from: https://guides.library.utoronto.ca/c.php?g=577919&p=4123572 by selecting the download link for the "How to Find & Document Grey Literature – Template".)

| **Date** | **Organization name & website URL**<br>*Ex. Healthy Ontario & http://www.behealthyontario.com/* | **Search strategy(s)/ words searched including (if applicable) how items were selected.**<br>*Ex. Used Advanced Google site/domain search*<br>*Search strategies:*<br>*1) Wind turbines and sleep*<br>*2) Wind-powered electrical generators and sleep*<br>*Selection:*<br>*All items retrieved in with each search were reviewed for relevance by 1 reviewer.* | **# items retrieved/ search results**<br>*Ex. Search Results:*<br>*1) 18*<br>*2) 15* | **# of items screened (uploaded to citation management software)**<br>*Ex. Items Screened:*<br>*1) 3*<br>*2) 2* |
|---|---|---|---|---|
| 07/28 | NIH<br>https://www.nih.gov/ | Artificial intelligence and indigenous | 29 | 2 |
| 07/28 | UNESCO<br>https://www.unesco.org/en | Artificial intelligence and indigenous (filtered to last 5 years, English, specific countries) | 56 | 1 |
| 07/28-9 | Gov't of Canada<br>https://www.canada.ca/en.html | Advanced search by specific terms: "indigenous" "artificial intelligence" "virtual health" "ethical use of AI" "responsible use of AI" | 571 | 6 |
| 07/29 | UK gov't<br>https://www.gov.uk/ | Indigenous artificial intelligence ethic (updated after 01/01/2020) [topics searched: business and industry; crime, justice and law; government; health and social care; regional and local gov't; society and culture; welfare; work] | 139 | 0 |
| 07/29 | US Dep't of State<br>https://www.state.gov/ | Responsible artificial intelligence | 106 | 0 |
| 07/29 | US Dep't of State: 2021-5<br>https://2021-2025.state.gov/ | "use of artificial intelligence" | 61 | 0 |
| 07/29 | US Dep't of State: 2017-21<br>https://2017-2021.state.gov/ | "use of artificial intelligence" | 6 | 0 |
| 07/29 | Australian Gov't: Department of Health, Disability and Ageing<br>https://www.health.gov.au/ | Responsible artificial intelligence | 52 | 0 |

| | | | | |
|---|---|---|---|---|
| 07/29 | Australian Gov't: Australian Institute of Health and Welfare https://www.aihw.gov.au/ | Artificial intelligence | 3 | 0 |
| 07/29 | NZ gov't https://www.govt.nz/ | artificial intelligence | 5 | 0 |
| 07/29 | CIHI https://www.cihi.ca/en | Artificial intelligence | 22 | 1 |
| 07/30 | NZ Ministry of Health https://www.health.govt.nz/ | Artificial intelligence | 6 | 1 |
| 07/30 | National Institute for Health and Care Excellence https://www.nice.org.uk/ | Artificial intelligence and ethic | 8 | 0 |
| | | Artificial intelligence and responsible | 18 | |

| **Date** | **Database name & URL** *Ex. ClinicalTrials.Gov & https://clinicaltrials.gov/* | **Search strategy(s)/ words searched including (if applicable) how items were selected.** *Ex. Search strategies:*<br>*1) Alzheimer's Disease and Canada.*<br>2) *Alzheimer Disease and Canada*<br>*Selection:*<br>*All results retrieved in the search were reviewed for relevance by 1 reviewer, 3 items were selected for screening.* | **# of items retrieved/ search results** *Ex. Search Results:*<br>*1) 181*<br>*2) 3* | **# of items screened (uploaded to citation management software)** *Ex. Items Screened:*<br>*1) 3*<br>*2) 0* |
|---|---|---|---|---|
| 07/30 | Preprints.org https://www.preprints.org/ | Artificial intelligence and health and ethical | 4 | 2 |
| 07/30 | Grey Matters | Artificial intelligence | 0 | 0 |
| 07/30 | OPHLA https://www.ophla.ca/p/index.html | Artificial intelligence | 0 | 0 |
| 07/30 | OAIster https://oaister.on.worldcat.org/discovery | kw:(artificial intelligence) AND kw:(telehealth) AND kw:(ethics) | 11 | 1 |
| 07/30 | Best Practice Guidelines https://rnao.ca/bpg/guidelines | "artificial intelligence" (filtering by EDI and health system; English language) | 11 | 0 |
| 07/30 | ProQuest Dissertations & Theses Global | "artificial intelligence" AND health AND ethic* [filters: English, last 5 years, | 54 | 1 |

| | | | | |
|---|---|---|---|---|
| | https://www.proquest.com/ | dissertations and theses] | | |

| **Date** | **Search engine**<br>*Ex. Google.ca* | **Search strategy(s) including how items were selected**<br>*Ex. Search strategies:*<br>*1) Wind turbines and sleep*<br>*2) Wind-powered electrical generators and sleep*<br>*Selection: Items were selected by scanning the first 100 results from each search* | **# of items screened (uploaded to citation management software)**<br>*Ex. Items Screened:*<br>*1) 3*<br>*2) 2* |
|---|---|---|---|
| 07/31 | Advanced Google<br>https://www.google.ca/advanced_search | Health and "artificial intelligence" AND (ethic or ethics or ethically or ethical or responsible) AND (Indigenous or Aboriginal or "First Nation" or Native or Amerindian or "American Indian" or "Alaska Native" or "Torres Strait Islander" or Maori) [filters: English, in text]<br>Items selected by scanning first 100 results | 15 |
